# Evaluation of Nutritional Status of Colorectal Cancer Patients from Algerian East Using Anthropometric Measurements and Laboratory Assessment

**DOI:** 10.18502/ijph.v49i7.3577

**Published:** 2020-07

**Authors:** Samira NEGRICHI, Salima TALEB

**Affiliations:** 1. Department of Living Beings Biology, University Larbi Tebessi, Tebessa, Algeria; 2. Laboratory of Water and Environment, University Larbi Tebessi, Tebessa, Algeria; 3. Department of Applied Biology, University Larbi Tebessi, Tebessa, Algeria; 4. Laboratory of Nutrition and Food Technology (LNTA), Institute of Nutrition and Food Technology (INATAA), University of Constantine, Constantine, Algeria

**Keywords:** Malnutrition, Nutritional deficiencies, Colorectal cancer, Algerian East

## Abstract

**Background:**

Chemotherapy may lead to cancer patient malnutrition, associated with reduced response, and increased complications to anticancer therapy. This study aimed to evaluate the nutritional status of Algerian colorectal cancer patients.

**Methods:**

This cross-sectional study was conducted during 2016–2017 at the Oncology departments from the Algerian East included 90 patients with colorectal cancer. For each patient, a questionnaire, anthropometric measurements and biochemical tests have been done.

**Results:**

The 50–59 yr and 60–69 age groups represented more than half of the population. Obesity and underweight were significantly higher in female patients according to their actual Body mass index (BMI). Prevalence of underweight increased after cancer diagnosis, while obesity prevalence has decreased significantly. The malnutrition classification based on Mid-upper-arm muscle circumference (MUAMC) and the triceps skinfold thickness (TST) has shown a significant higher prevalence of malnutrition among male patients than females. The classification of Nutrition risk index (NRI) has shown a significant high percentage of male patients having malnutrition. In our study, no significant differences were recorded for biochemical tests. Anemia recorded the higher prevalences for both sex compared to other deficiency. Hypoironemia prevalence’s was higher among male patients than females while hypokalemia and hypoproteinemia prevalence’s were higher in female patients.

**Conclusion:**

Malnutrition in CRC patients must be combated by early detection to decrease complications associated to cancer and chemotherapy.

## Introduction

By its frequency, colorectal cancer (CRC) is the third most common malignancies worldwide (1) compared to industrialized countries, CRC incidence is lower in Algeria, and the estimated number for both sexes is nearly 5000 new cases and the numbers are growing unstoppably (2). CRC comes in second position after lung cancer in men and after breast cancer in women (2). It is a multifactorial disease; it can be caused by non-modifiable risk factors such as heredity, and/or compartmental risk factors and modified such as lifestyle habits (3). Lifestyle and diet during CRC treatment are much less known than in the prevention (4). Thus, among the malnourished of all patient groups, cancer patients are the most affected ones (5) by malnutrition; a state in which the nutritional status is altered and the “most common co-morbidity in cancer patients”(6) which can affect 30% to 60% patient of CRC (7). Malnutrition has a negative impact on the response to therapy and the patient’s quality of life. It can be caused by the tumor itself, the patient response to the tumor and the anticancer therapies (6). Unfortunately, in most oncology units, malnutrition is taken too lightly (8) and colorectal cancer-related malnutrition can have serious consequences when it is not treated, and the patient’s tolerance of chemotherapy cycles decreases (9). Chemotherapy uses chemical substances that do not act strictly locally to destroy cancer cells (10). It may cause different side effects, such as nausea and vomiting (11). As well, it may directly alter the taste perception, which may lead to patient malnutrition (12).

Obesity is one of the most prevalent risk factors for CRC and different diseases (13). Obese or underweight CRC patients may experience higher mortality rates than normal and overweight patients, which make obesity a risk factor before and after CRC diagnosis (14). For that, an early nutritional intervention to overweight and mal-nourished patients; would provide a better prognosis, and decrease rates of morbidity and mortality among cancer patients (15).

Due to the economic, sociologic and epidemiologic consequences of CRC related malnutrition particularly in Algeria and generally in the world, the present study focus on the evaluation of the nutritional status of CRC patients undergoing chemotherapy using nutritional assessment and laboratory markers; at five oncology departments in the Algerian East.

## Methods

### Population presentation

This study was conducted between Apr 2016 and Dec 2017 at Oncology departments of multiple “Centers for Cancer Control” from the Algerian East, treating almost all types of cancer. These Oncology departments were in Annaba, Batna, Constantine, Setif and Tebessa.

The survey included 90 subjects of all ages and all stages of CRC. Thirty-seven women and 53 men, who had a confirmed diagnosis of CRC (ICD codes C18–20) admitted for chemotherapy, were included in this study. Oxaliplatin, cetuximab, bevacizumab and capecitabine were the most common CRC medications given to treat the patients. We obtained the consent of all patients for participation in this study. We also obtained the consent of each patient for the collection of a blood sample.

The questionnaire was completed for each patient using a face-to-face interview, to avoid any misunderstand of questions or information’s lack. All the data collected during each interview and the results of anthropometric measurements and biochemical tests were registered on an individual record survey. Sociodemographic information included age, sex, marital status, education level, body weight and height, history of body weight, smoking habits, alcohol habits, history of medication (consumption of NSAIDs), family history of cancer and address; while clinical information included the cancer staging and CRC topography.

### Biochemical assay and anthropometric measurements

Patients underwent fasted blood draws by veni-puncture, and the samples were collected in EDTA tube (purple top tube) for hematology tests, and plain tube (red top tube) for serum biochemistry tests. Complete blood count was realized using an automatic hematology analyzer “Mindray BC 5300” while serum concentration of albumin, total protein, iron and potassium; were measured using an automatic biochemistry analyzer “Mindray BS-200”. Transferring serum concentration was measured using an analyzer “ARCHITECT ci-4100”.

A mechanical scale “beurer-MS01” was used to measure the weight in kilogram (kg), and a height chart “micro-toise PSCC” for length measure in meter (m), a tape measure to measure the mid-upper-arm circumference (MUAC) in centimeters (cm) and a skinfold caliper to measure the triceps skinfold (TSF) thickness in millimeters (mm). Normal values of the triceps skinfold thickness, are between 12–13mm in men and 16–17mm in women (16). Patients were asked about their body weight before CRC diagnosis.

Body Mass Index (BMI) was calculated as: weight (kg) divided by height (m^2^). Four groups classification was used: Underweight: BMI <18.5 kg/m^2^. Normal: 18.5<BMI<24.99 kg/m^2^. Overweight: 25<BMI<29.99 kg/m^2^ and Obese: BMI≥30 kg/m^2^ (17). Percentage of weight change was calculated as ((usual weight-actual weight)/usual weight) x100. Weight loss greater than 10% indicates malnutrition, above 25% indicates severe malnutrition (18). The Nutritional Risk Index was calculated as NRI = 1.519 × serum albumin (g/l) + (41.7× (current weight/usual weight)). If NRI>100 there is no nutrition risk, and there is a borderline nutrition risk if: 97.50 ≤ NRI ≤ 100, a mild nutrition risk: NRI 83.50 ≤ NRI ≤97.5 and severe nutritional risk: NRI <83.5(18).The mid-upper-arm muscle circumference (MUAMC) was calculated by the formula: MUAMC = MUAC – (0.314 × TSF), malnutrition is defined when MUAMC< 19cm in females patients, while it is defined in males when MUAMC < 24 cm form males < 65 yr, and < 22 cm form males > 65 yr (16).

### Statistical analysis

Results are expressed as percentages (categorical variables), mean and standard deviation SD (Continuous variables). We used Chi-square tests to compare the percentages of parameters we surveyed between female and male patients. The student t-test was used to compare means of age, weight, BMI, Mid-upper-arm circumference, Triceps skinfold thickness and various biochemical parameters. A *P*-value<0.05 was considered to be statistically significant. Statistical processing of the results was performed using Minitab ver.13.

### Limitations

- Realization of blood samples was not allowed in some departments.
- Some patient care refused to carry out the questionnaire with their relatives, and patients were excluded from the study.
- The expensive cost of reagents forced us not to do certain parameters (vitamins and trace elements).

### Ethical Approval

Informed consent was obtained from all participants in the survey. The study follows the ethical standards of the Helsinki Declaration (2013 revision).

## Results

### Patient demographics and tumor characterization

Ninety CRC patients admitted for chemotherapy were included in this study (53 men and 37 women); 55% of them are aged between 50 to 69 yr old (Table1) and the mean age of males was significantly higher than the mean age of females patients (60 vs. 51 yr old; *P*=0.001) (Table2). Colon cancer was more frequent than other localizations; colon cancer was also more frequent among females, while rectal cancer was more frequent in males. In this study, 70% of the population was in the IV stage. The mean BMI was significantly higher in the female patients’ than in the male’s (*P*=0.034) before and after cancer diagnosis (*P*=0.036 and 0.034 respectively). Mean triceps skinfold thickness was significantly higher in females patients than males (*P*=0.000) (Table 2).

**Table 1:** General characteristics of study population

***Variable***	***Female n (%)***	***Male n (%)***	***Total n (%)***	***P-value***
Sex	37 (41.11)	53 (58.89)	90 (100)	
Age group(yr)				
30 – 39	7 (18.92)	4 (7.55)	11 (12.22)	0.105
40 – 49	10 (27.03)	3 (5.66)	13 (14.44)	0.005
50 – 59	9 (24.32)	17 (32.08)	26 (28,.89)	0.425
60 – 69	7 (18.92)	17 (32.08)	24 (26,.67)	0.165
70 – 79	4 (10.81)	10 (18.87)	14 (15.56)	0.299
80 – 89	0 (0)	2 (3.77)	2 (2.22)	1.428
Localization				
Colon	29 (78.38)	33 (62.26)	62 (68.89)	0.104
Recto-sigmoid	3 (8.11)	5 (9.43)	8 (8.89)	0.828
junction	5 (13.51)	15 (28.3)	20 (22.22)	0.097
Rectum				
Stage				
I	2 (5.41)	1 (1.89)	3 (3.33)	0.360
II	7 (18.92)	9 (16.98)	16 (17.78)	0.813
III	4 (10.81)	4 (7.55)	8 (8.89)	0.592
IV	24 (64.68)	39 (73.58)	63 (70)	0.374

(n: effective)

**Table 2:** Mean value ± SD of nutritional parameters of the study population

***Variables***	***Mean Value ± SD***		***P-value***	***Reference value (19,20)***
	***Female***	***Male***		
Age (yr)	51.97 ± 12.48	60.25 ± 11.06	0.001	
Anthropometric measurements				
Weight (kg)	61.91 ± 15.4	64.19 ± 12.00	0.431	
BMI before cancer (kg/m^2^)	28.44 ± 5.57	26.23 ± 4.28	0.036	
Actual BMI (kg/m^2^)	25.09 ± 6.13	22.89 ± 3.56	0.034	
Mid-upper-arm circumference	26.82 ± 5.00	25.32 ± 3.35	0.091	
Triceps skinfold thickness	18.16 ± 8.48	9.43 ± 4.53	0.000	
Laboratory markers				
Transferrin (g/L)	2.58 ± 0.61	2.86 ± 0.60	0.488	2–3.2 g/l
Serum iron (μg/dL)	45.85 ± 20.89	53.89 ± 21.87	0.087	Males 65–175; Females 40–150 μg/dl
Potassium (mmol/l)	3.66 ± 0.59	3.81 ± 0.57	0.229	3.5–4.5 mmol/l
Albumin (g/L)	36.48 ± 6.14	36.94 ± 5.84	0.716	35–50 g/l
Total protein (g/L)	61.07 ± 15.67	61.36 ± 13.19	0.926	55–80 g/l
Hemoglobin (g/dL)	11.38 ± 1.46	11.32 ± 2.21	0.893	12–16 g/dl

(*P*-value obtained from Student *t*-test)

Mean concentration of transferrin, albumin, total protein, calcium and potassium in male and female patient were among normal values, but no significant differences were found (*P*=0.488; 0.229; 0.716; 0.191; 0.229 respectively). Mean concentration of iron was below the normal values in male patients but among normal values in females (0.087). The mean concentrations of hemoglobin were below the normal values for both sexes (*P*=0.893).

**Fig. 1:**
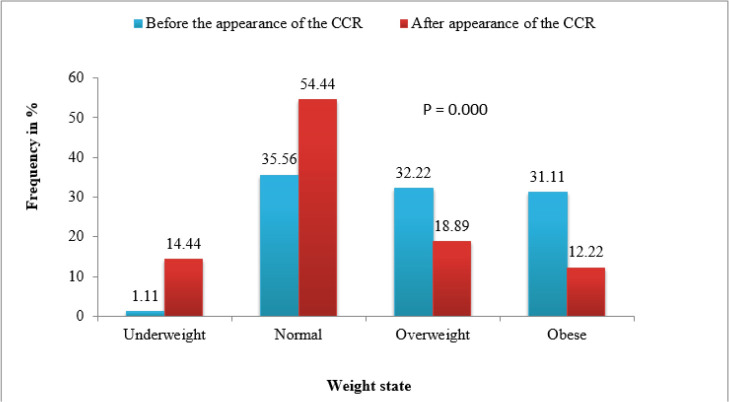
Population distribution according to BMI before and after CRC appearance

In our study, the percentage of underweight went from 1% to 14%; thus obesity and overweight prevalences decreased from 31% and 32% before CRC diagnosis to 12% and 18% after the CRC appearance; respectively (P=0.000) (Fig. 1).

**Fig. 2:**
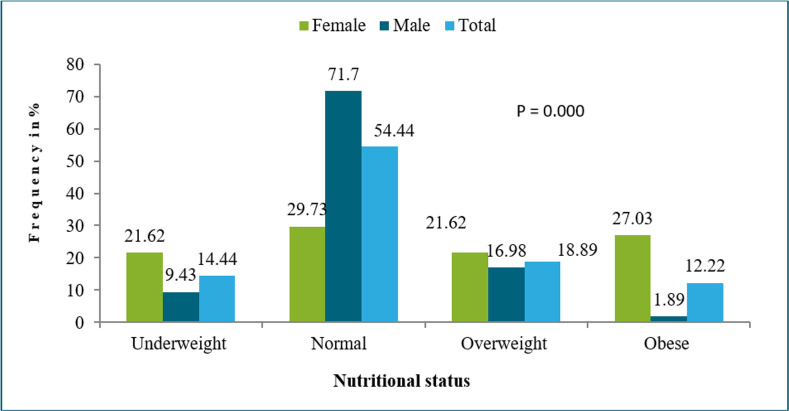
Population distribution by sex according to the BMI

Obesity and underweight were higher in female patients more than males; 71% of male patients have normal BMI vs 29% of females. Under-weight was more frequent in females than in males (21% vs 9%) and the difference was significant (p=0.000) (Fig. 2).

Based on the percentage weight loss, 43.24% females and 35.85% males had moderate malnutrition (*P*=0.479). The malnutrition classification based on Mid-upper-arm muscle circumference has shown a significantly higher prevalence of malnutrition among male patients than females (60.38% vs 24.32%, *P*=0.001), the same result was obtained using the classification based on the triceps skinfold thickness (71.7% vs 40.54%, *P*=0.003). The classification of NRI has shown that percentage of females in severe malnutrition range was higher than males (24.32% vs 9.43%; *P*=0.055), while the percentage in malnutrition range was higher in male patients (62.26% vs 40.54%; *P*=0.042) (Table 3).

**Table 3:** Evaluation of the population nutritional status based on nutritional assessment

***Malnutrition based on***	***Female n (%)***	***Male n (%)***	***Total n (%)***	**P-*value***	***Reference value***
Weight loss percentage				0.715	
Normal	15 (40.54)	26 (49.06)	41 (45.56)	0.425	
Moderate malnutrition	16 (43.24)	19 (35.85)	35 (38.89)	0.479	> 10%
Severe malnutrition	6 (16.22)	8 (15.09)	14 (15.56)	0.885	> 25%
Mid-upper-arm muscle circumference				0.001	
Normal	28 (75.68)	21 (39.62)	49 (54.44)	0.001	Females < 19 cm
Malnutrition	9 (24.32)	32 (60.38)	41 (45.56)	0.001	males < 24 cm : age < 65 < 22 cm: age > 65 years
Triceps skinfold thickness				0.002	
Higher than standards	19 (51.35)	9 (16.98)	28 (31.11)	0.001	
Normal	3 (8.11)	6 (11.32)	9 (10.00)	0.617	Males:12–13 mm
Malnutrition	15 (40.54)	38 (71.7)	53 (58.89)	0.003	Females:16–17 mm
Classification of NRI				0.126	
Normal	10 (27.03)	10 (18.87)	20 (22.22)	0.360	>100
Risk of malnutrition	3 (8.11)	5 (9.43)	8 (8.89)	0.828	97.50 ≤ NRI ≤ 100
Malnutrition	15 (40.54)	33 (62.26)	48 (53.33)	0.042	NRI 83.50 ≤NRI≤97.5
Severe malnutrition	9 (24.32)	5 (9.43)	14 (15.56)	0.055	NRI <83.5

(n: effective, %: prevalence)

As shown in Table 4 mild degree of protein malnutrition and severe depletion were higher among female patients than males according to the serum transferrin levels classification; while moderate depletion was more frequent among male patients (*P*=0.689).

**Table 4:** Evaluation of the population nutritional status based on laboratory markers

***Malnutrition based on***	***Female n (%)***	***Male n (%)***	***Total n (%)***	**P-*value***	***Reference value (19,20)***
	Serum transferrin levels				
Standard	32 (86.49)	46 (86.79)	78 (86.67)	0.966	2–3.2 g/L
Mild degree of protein malnutrition	2 (5.41)	5 (9.43)	7 (7.78)	0.483	1.5–2 g/L
Moderate depletion	2 (5.41)	2 (3.77)	4 (4.44)	0.689	1–1.5 g/L
Severe depletion	1 (2.70)	0 (0.00)	1 (1.11)	-	>1 g/L
	Serum iron concentration				
Standard	18 (50.00)	16 (30.77)	34 (38.64)	0.076	Males 65–175
Iron deficiency	18 (50.00)	36 (69.23)	54 (61.36)	0.066	μg/dL; Females 40–150 μg/dL
	Albumin				
Standard	24 (64.86)	35 (66.04)	59 (65.56)	0.908	35–50 g/l
Moderate malnutrition	7 (18.92)	14 (26.42)	21 (23.33)	0.408	30–35 g/l
Severe malnutrition	6 (16.22)	4 (7.55)	10 (11.11)	0.198	< 30 g/l
	Serum Total protein				
Hypoproteinemia	14 (37.84)	14 (26.42)	28 (31.11)	0.249	< 55 g/L
Standard	19 (51.53)	37 (69.81)	56 (62.22)	0.076	55–80 g/L
Hyperproteinemia	4 (10.81)	2 (3.77)	6 (6.67)	0.188	> 80 g/L
	Serum Potassium level				
Standard	24 (64.86)	41 (77.36)	65 (72.22)	0.193	3.5–4.5 mmol/L
Mild hypokalemia	10 (27.03)	11 (20.75)	21 (23.33)	0.489	3.0–3.5 mmol/L
Severe hypokalemia	3 (8.11)	1 (1.89)	4 (4.44)	0.159	< 2.5 mmol/L
	Hemoglobin levels				
Anemia	26 (70.27)	32 (60.38)	58 (64.44)	0.335	< 12g/dL
Standard	11 (29.73)	20 (37.74)	31 (34.44)	0.432	12–16 g/dL
Hyper-hemoglobinemia	0 (0.00)	1 (1.89)	1 (1.11)	0.706	> 16g/dL

(n: effective, %: prevalence)

Iron deficiency was frequent with a prevalence of 61%, it was more frequent in males than females (*P*=0.066). Moderate malnutrition based on serum albumin levels classifications was higher in males while severe malnutrition was higher in females (*P*=0.408; *P*=0.198 respectively). About 31% of patients suffer from hypoproteinemia, and the percentage was higher in female patients (*P*=0.249). Moreover, mild and severe hypokalemia were higher among female patients, but no significant differences were found (*P*=0.489; *P*=0.159 respectively). We used hemoglobin levels to determine anemia, which was very frequent among our patients (64%) and higher in females than males (*P*=0.335).

## Discussion

The mean age of the studied subjects was significantly lower in female patients than in males, and the most affected age groups of females are younger than male patient’s most affected age groups. These findings are consistent with another Algerian study (2). Colon cancer was more frequent than rectal cancer; it was also more common among women, while rectal cancer was more common among men. These results are consistent with literature findings (21, 22).

Obesity was significantly more frequent before CRC diagnosis, despite the decrease in obesity and over-weight prevalence’s after CRC appearance, the frequencies still high.

These finding is consistent with Renate study (23), in which, values are close to our population study. Our results confirm the association between CRC chemotherapy and weight loss, using patients BMI, the prevalence of underweight known an increase after CRC appearance. This change was highly significant (*P*=0.000); and female patients were more affected than male patients were (*P*=0.000). A study conducted by Tolentino reported a similar result (24). Using other Nutritional status assessment, such as: MUAMC and TST shown that malnutrition was frequent among CRC patients, especially among male patients, and this results is due to the presence of receptors of epinephrine alpha in large numbers in thigh regions in females, these receptors inhibit lipolysis which explains the higher percentage of body fat in females than in males (25). This result is similar to the results found in an Indian study (26). Using NRI showed a higher percentage of moderate malnutrition among males, and a higher percentage of severe malnutrition among females. Hypoalbuminemia results from an inadequate caloric intake, which explains the high prevalence of malnutrition among CRC patients.

BMI class change from normal to underweight was higher for gastrointestinal than other malignancies (24; 27). In a recent report, certain chemotherapeutic agents may cause weight loss in obese mice by depleting stores of adipocytes (28).

The association of weight loss was proved with CRC; this malnutrition was related to eating disorders, anorexia, chemotherapy toxicity and nutritional effects of CRC (29).

Hypokalemia and hypoproteinemia are high in our population; and may be caused by neoplasia and inadequate dietary intake, extrarenal losses and redistribution abnormalities (30). The mean values of potassium in our population study are lower than these found in Chinese (31).

Serum transferrin is a useful nutritional marker. Patients with a serum transferrin level below the reference range had most of the major life threatening complications and deaths (32). Iron is transported by transferring to all body tissues including bone marrow. Even if iron reserves are normal, tissues go short of bioavailable iron, when patients are treated with some chemotherapeutics agents that stimulate erythropoiesis (33). More than half of patients had serum Iron and hemoglobin level below the reference values. About a third of patients had albumin and total protein levels below the reference values. The hemoglobin and albumin mean values of this survey are consistent with these of a recent polish study (34).

A Japanese study showed that low serum iron levels in CRC patient are related to the size and the bleeding of the adenoma causing iron deficiency (35) which is a frequent complication in CRC patients and one of the most common anemia causes (36).

In the European Cancer Anemia Survey, 67% had anemia during chemotherapy (37), this result is very close to anemia prevalence in this study (64%). CRC is the cause of iron deficiency and iron deficiency-associated anemia (38), these deficiencies are the consequence of the cancer-associated cytokine release, what make theme common complications in patients (39).

A high prevalence of CRC patients in our study were identified as malnourished by the hypoalbuminemia, our prevalence is higher compared to an American study (40).

Albumin serum levels, reflects the systemic inflammatory response as well the loss of lean tissue (41, 42). Cancer cells can produce cytokines that modulates the production of albumin (43). As well, different cancer drugs cause low serum albumin (44).

## Conclusion

In Algeria, CRC related-malnutrition is a serious health problem, and that was confirmed in this study. Malnutrition must be combated by early detection to improve the daily quality of life and decrease morbi-mortality rates among cancer patients.

## Ethical considerations

Ethical issues (Including plagiarism, informed consent, misconduct, data fabrication and/or falsification, double publication and/or submission, redundancy, etc.) have been completely observed by the authors.
